# p53 Stabilization Induces Cell Growth Inhibition and Affects *IGF2* Pathway in Response to Radiotherapy in Adrenocortical Cancer Cells

**DOI:** 10.1371/journal.pone.0045129

**Published:** 2012-09-19

**Authors:** Camilla Sampaoli, Lidia Cerquetti, Randa El Gawhary, Barbara Bucci, Donatella Amendola, Rodolfo Marchese, Silvia Misiti, Giuseppe Novelli, Vincenzo Toscano, Antonio Stigliano

**Affiliations:** 1 Endocrinology, Department of Clinical and Molecular Medicine, Sant'Andrea Hospital, Faculty of Medicine and Psychology “Sapienza” University of Rome, Rome, Italy; 2 Research Center, San Pietro Hospital, Rome, Italy; 3 Radiation Oncology Unit, San Pietro Hospital, Rome, Italy; 4 Department of Experimental Medicine, “Sapienza” University of Rome, Rome, Italy; Ospedale Pediatrico Bambino Gesu', Italy

## Abstract

Adrenocortical carcinoma (ACC) is a very rare endocrine tumour, with variable prognosis, depending on tumour stage and time of diagnosis. However, it is generally fatal, with an overall survival of 5 years from detection. Radiotherapy usefulness for ACC treatment has been widely debated and seems to be dependent on molecular alterations, which in turn lead to increased radio-resistance. Many studies have shown that p53 loss is an important risk factor for malignant adrenocortical tumour onset and it has been reported that somatic mutations in *TP53* gene occur in 27 to 70% of adult sporadic ACCs. In this study, we investigated the role of somatic mutations of the *TP53* gene in response to ionizing radiation (IR). We studied the status of p53 in two adrenocortical cell lines, H295R and SW-13, harbouring non-functioning forms of this protein, owing to the lack of exons 8 and 9 and a point mutation in exon 6, respectively. Moreover, these cell lines show high levels of p-Akt and *IGF2*, especially H295R. We noticed that restoration of p53 activity led to inhibition of growth after transient transfection of cells with wild type p53. Evaluation of their response to IR in terms of cell proliferation and viability was determined by means of cell count and TUNEL assay.^wt^p53 over-expression also increased cell death by apoptosis following radiation in both cell lines. Moreover, RT-PCR and Western blotting analysis of some p53 target genes, such as *BCL2*, *IGF2* and Akt demonstrated that p53 activation following IR led to a decrease in *IGF2* expression. This was associated with a reduction in the active form of Akt. Taken together, these results highlight the role of p53 in response to radiation of ACC cell lines, suggesting its importance as a predictive factor for radiotherapy in malignant adrenocortical tumours cases.

## Introduction

Adrenocortical carcinoma (ACC) is a very rare endocrine tumour, with an incidence estimated at approximately 1 to 2 cases *per* 1 million people every year in the general adult population [1], [2], while in the infant population of Southern Brazil the frequency of this malignancy is relatively high, ranging from 3,4 to 4,2 *per* million children [3], [4]. Statistical age distribution follows a bimodal trend, with a first peak occurring in early childhood and a second one in the fourth and fifth decades of life [2], [5].

ACCs show variable prognosis, depending on tumour stage and time of diagnosis, although they are generally fatal, with an overall survival of 5 years from detection [6]. Frequency of metastasis associated with ACC varies depending on the study, ranging from 30% to 85% of patients with distant metastasis at the time of presentation [7].

Currently, the therapy that seems to produce best results in ACC cases is surgical resection of the adrenal mass. However, postoperative disease-free survival at 5 years is only around 30% and recurrence rates are up to 85%. Medical therapy with o,p'DDD (ortho, para', dichloro-, diphenyl-, dichloroethane, or mitotane) and other cytotoxic drugs is limited by important side effects. Therefore, other adjuvant treatment options after complete tumour removal are needed [8], [9].

Radiotherapy has often been considered ineffective for ACC treatment. In fact, up to 58% of patients do not show a significant response. Although data collected from patients treated with adjuvant tumour bed irradiation demonstrated that radiotherapy may be effective in reducing the high rate of local recurrence of ACC, the high variability in the observed effects suggests that more surveys are needed in order to fully understand the effective therapeutic role of radiotherapy [8], [10]. At present, it is thought that radiotherapy should be individualized, while taking into account parameters such as tumour size, resection status, Ki-67 index and tumour spread [11].

Moreover, the variability of the responses observed highlighted the importance of identification of genetic and molecular alterations involved in the resistance of some tumours to radiotherapy [2], [12]. At present, knowledge of genetic alterations leading to ACC predisposition is quite poor. However, many studies have shown that p53 loss is an important risk factor for malignant adrenocortical tumour onset. Patients affected by Li-Fraumeni syndrome, who inherit germline mutations of *TP53*, are more likely to develop malignant adrenocortical tumours. Moreover, a specific mutation in codon 337 (R337H) seems to be responsible for the majority of cases of ACC in the child population of Southern Brazil. Furthermore, it has been also reported that somatic mutations in *TP53* gene occur in 25 to 70% of adult sporadic ACCs [2],[9].

In this study, we investigated the role of p53 in response to ionizing radiation (IR) in ACC *in vitro* models. Specifically, we analysed the status of *TP53* gene in two human ACC cell lines, H295R and SW-13, and we found that this gene is mutated in both cell lines, which are partially resistant to IR at a dose of 6 Gy, as previously demonstrated [13]. The over-expression of *IGF2* mRNA is another typical feature of ACC found in H295R cell line. Here, we showed that the restoration of p53 activity, by transfection of wild type p53 (^wt^p53), led to inhibition of growth and induced cell death by apoptosis following IR administration in both cell lines. We also noticed a reliable decrease in *IGF2* gene expression and a reduction in Akt activation following IR treatment in cells over-expressing wild type p53.

Taken together, these results highlight the importance of p53 in cellular response to IR in ACC cell lines. They also indicate a molecular mechanism involving IGF2, thus strengthening the effectiveness of radiotherapy as adjuvant treatment in ACC.

## Results

### *TP53* mRNA analysis

**Figure 1:**
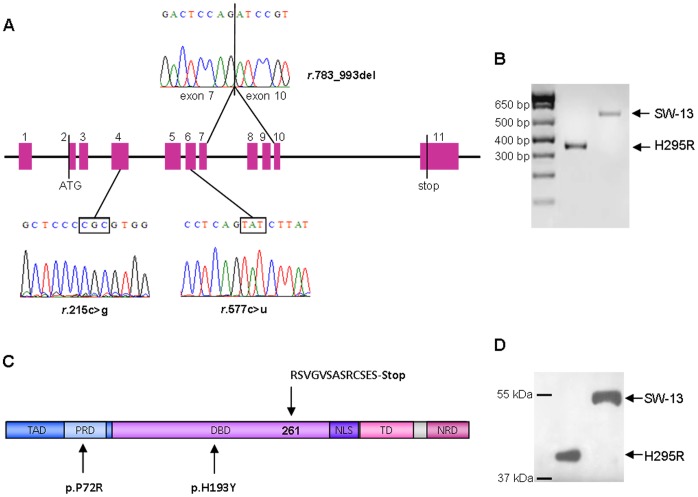
Sequencing analysis of *TP53* coding sequence in H295R and SW-13 cell lines. (A) Representation of *TP53* mutations identified in H295R and SW-13 cell lines, showing a large deletion affecting 211 base pairs in H295R cells (top) and a homozygous point mutation in exon 6 (*r.577c>u*) in SW-13 cell line (bottom). SW-13 cells also exhibit a polymorphic site located in exon 4 (*r.215c>g*). (B) Inverted image relative to RT-PCR analysis of exons 7 to 10 of *TP53* gene. Electrophoresis on a 2% agarose gel revealed a major band of ∼575 bp, corresponding to SW-13 transcript, and a minor band of ∼364 bp, corresponding to H295R transcript (DNA ladder, 1 Kb plus). (C) Predicted amino-acid sequence of p53 in H295R and SW-13 cell lines. Deletion of exons 8 and 9 in H295R determines a frameshift starting at codon 261, creating a subsequent stop codon at position 274 (top). In SW-13 cells, a homozygous point mutation at codon 193 determines an amino-acid substitution (histidine to tyrosine) in the DBD of the protein (bottom). (D) Western blotting analysis of p53 in H295R and SW-13 cells, showing the presence of a shorter protein in H295R cell line, which molecular weight is ∼44 kDa.

In a previous paper by our group [13], a large deletion in *TP53* gene, affecting exons 8 and 9, was discovered in H295R cell line. We performed sequencing analysis of *TP53* coding sequence in these cells to evaluate the effect of this deletion on mRNA production. We found that a shorter mRNA is transcribed, where exon 7 is directly bound to exon 10 (Figure 1, panel A, top). Consistent with that, RT-PCR analysis of *TP53*, was carried out using primers flanking exons 8 and 9 region. This revealed a shorter mRNA product, lacking 211 base pairs (Figure 1, panel B).

The deletion of exons 8 and 9 determines an amino acid sequence change starting from codon 261, which also introduces a premature stop codon at amino acid position 274 (Figure 1, panel C, top). According to our previous data, this mRNA was shown to be translated into a shorter protein, whose molecular weight is about 44 kDa (Figure 1, panel D).

Sequencing analysis of *TP53* coding sequence in SW-13 cells confirmed the presence of a homozygous point mutation in exon 6 (*r.577c>u*), which was described for the first time by Reincke *et al.* [14] (Figure 1, panel A, bottom). This results in amino acid change at codon 193 (histidine to tyrosine) (Figure 1, panel C, bottom). Moreover, we identified a polymorphism in codon 72 (CCC to CGC) (Figure 1, panel A, bottom) in this cell line, resulting in an amino acid substitution at this position (arginine instead of proline) (Figure 1, panel C, bottom). Resulting p53 protein in these cells has a standard molecular weight (Figure 1, panel D) and is highly expressed compared with LNCaP cell line, which harbours the wild type form of p53, as was demonstrated by Western blotting analysis (Figure S1).

### Effect of wild type *TP53* on cell proliferation and response to ionizing radiation

To assess the significance of *TP53* mutations on cell proliferation, we transiently transfected H295R and SW-13 cells with empty vector (mock) or a vector expressing the wild type form of *TP53* (WT) and evaluated the effect on cell growth 24, 48 and 72 hours after transfection. We also evaluated the effect of *TP53* restoration in response to irradiation at dose of 6 Gy.

The same analysis was performed on radio-resistant ovarian adenocarcinoma SK-OV-3 cells, which do not express either p53 protein or mRNA [15].

**Figure 2:**
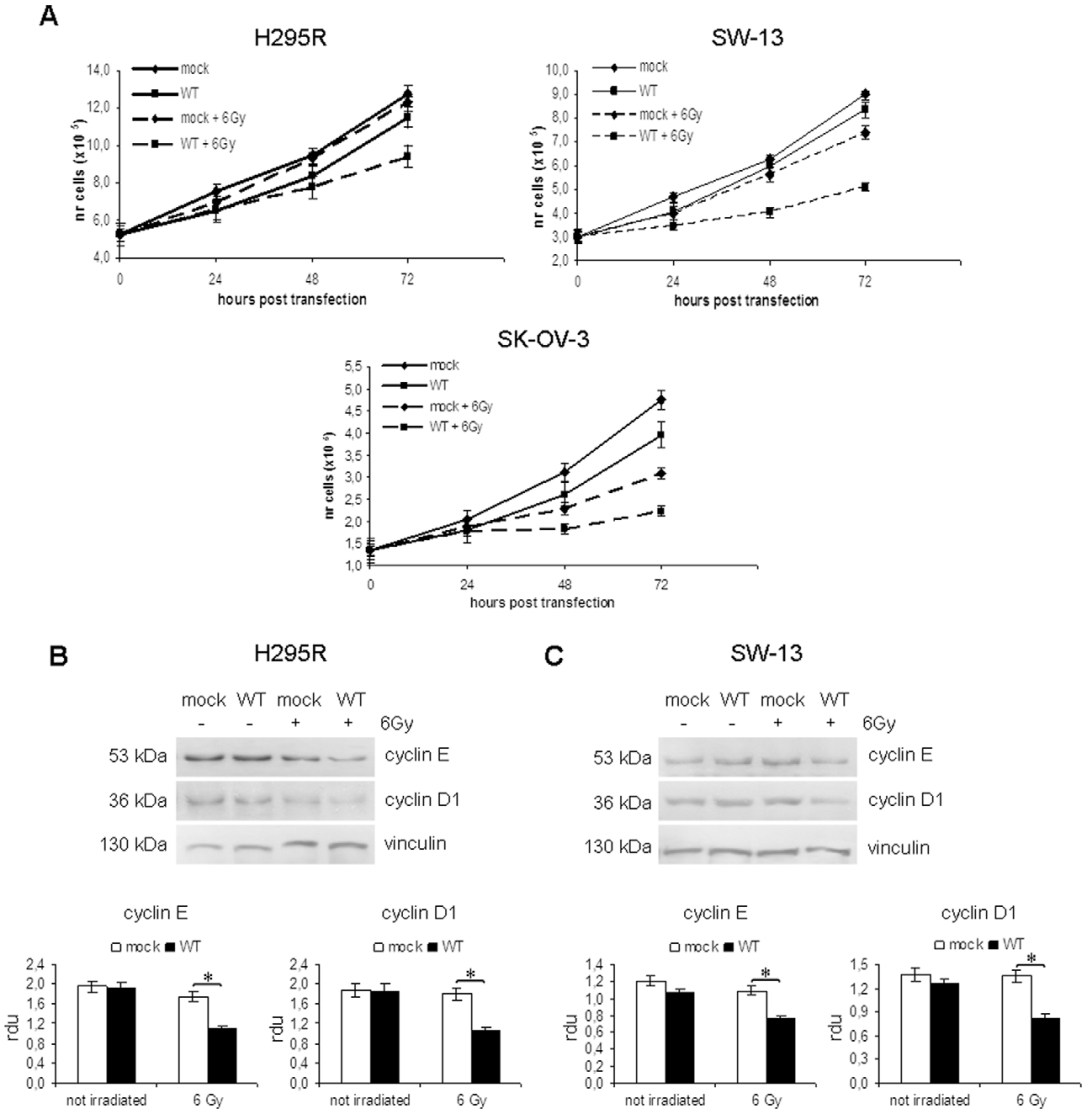
Effect of p53 over-expression and irradiation on cell proliferation in H295R, SW-13 and SK-OV-3 cells. Cell growth was evaluated in H295R, SW-13 and SK-OV-3 cell lines transfected with empty vector (mock) or p53-vector (WT), either irradiated or not. (A) In H295R cell lines (left), ionizing radiation (IR) treatment induces a significant inhibition of cell growth in cells expressing wild type p53, starting from 48 h after transfection (−18% compared to mock irradiated cells; p<0.05) and reaching −25% after 72 h (p<0.01). Irradiation does not induce a stable effect in SW-13 cells (right) transfected with empty vector, whereas cells expressing wild type p53 are significantly inhibited at 48 h after transfection (−28%; p<0.05) until the end of the experiment (−31%; p<0.01). In SK-OV-3 cells (bottom), p53 restoration induces an strong effect on cell growth, which is evident since 48 from transfection (−20% of growth inhibition in WT+IR sample compared with mock +IR; p<0.01) and endures up to 72 h (−28%; p<0.01). Data are mean ± S.D. of at least three independent experiments carried out by duplicates. (B) Western blotting analysis of cyclin E and cyclin D1 expression in H295R cells at 72 h after transfection revealed a down-regulation of these proteins in cells expressing ^wt^p53 treated with IR at a dose of 6 Gy. (C) Analysis of cyclin E and cyclin D1 expression in SW-13 cell line by Western blotting shows a decrease in protein levels in samples transfected with p53-vector after irradiation. Bands' intensities were quantified with ImageJ software, using vinculin for normalization. (*, p<0.05).

In H295R cells (Figure 2, panel A, left), no significant growth inhibition was observed in cells expressing wild type p53 compared with cells transfected with empty vector (−11% after 48 h and −9% at 72 h). This indicates that p53 alone is not able to induce a stable growth arrest in these cells. IR did not significantly alter cell proliferation in cells transfected with empty vector (−4% after 72 h), whereas the effect of irradiation on cell growth was evident in cells expressing ^wt^p53. This effect was evident at 48 h after transfection (−18% compared to mock irradiated cells; p<0.05) and strengthened at 72 h after transfection (48 h after irradiation), when growth inhibition reached −25% (p<0.01).

Unlike our previous report [13], in this work we improved transfection efficiency by using pBABE-neo-p53 vector, containing the MMLV Long Terminal Repeat. Morgenstern and Land [16] support the ability of this vector to greatly improve gene expression in many cell lines.

Similar results were observed in SW-13 cell line (Figure 2, panel A, right). Cells transfected with empty vector or p53-vector showed a very similar proliferation trend. Moreover, irradiation did not significantly affect cell proliferation in cells transfected with empty vector. On the contrary, a strong effect of IR treatment in cells expressing ^wt^p53 was evident at 24 h after irradiation (48 h after transfection), with 28% of cell growth inhibition (p<0.05) compared with irradiated cells transfected with empty vector. The effect was even greater at 72 h after transfection, when we observed 31% of growth inhibition in WT irradiated cells compared with control (p<0.01).

In SK-OV-3 cells (Figure 2, panel A, bottom), the restoration of ^wt^p53 induced a slight inhibition of cell growth, which however did not exceed 17% after 72 h. We also observed that IR treatment at a dose of 6 Gy induced a growth inhibition of −27% (p<0.05) after 24 h in cells transfected with empty vector, which reached −35% (p<0.01) after 48 h. However, the presence of ^wt^p53 strengthened the effect of IR treatment. In fact, we observed a cell growth inhibition of −20% (p<0.01) 24 h after irradiation in cells expressing p53 compared with those transfected with empty vector. This effect increased after 48 h (−28%; p<0.01).

The importance of p53 in SK-OV-3 cells response to irradiation was further confirmed by the observation that cells transfected with empty vector began to recover 72 h after IR treatment, while those expressing ^wt^p53 continued to be inhibited (data not shown).

In H295R and SW-13 cell lines, the effect of p53 stabilization on cyclin E and cyclin D1 was evaluated, as these proteins contribute to cell proliferation by accelerating the G1 phase of cell cycle [17], [18]. At 72 h after transfection with empty vector (mock) or p53 vector (WT), we observed a decrease in cyclin E and cyclin D1 expression levels in samples expressing ^wt^p53 irradiated with a dose of 6 Gy, in both H295R (Figure 2, panel B) and SW-13 (Figure 2, panel C) cells. This result hence indicates that IR treatment is able to slow the proliferation rate of our cell lines in a p53-dependent manner.

These data indicate that restoration of ^wt^p53 expression in H295R and SW-13 cell lines is not sufficient to exert an effect on cell proliferation. The presence of the wild type form of p53 appears to be fundamental for growth inhibition of these cells in response to irradiation, as cells transfected with empty vector are hardly affected by this treatment. However, ^wt^p53-expressing cells are significantly inhibited.

In SK-OV-3 cells, the effect of IR is stronger than in H295R and SW-13 cells, even in samples transfected with empty vector, suggesting that this cell line is more sensitive to irradiation than ACC cell lines. Nevertheless, the maximum growth inhibition is reached when the wild type form of p53 is expressed, indicating that this protein is involved in the response to IR in SK-OV-3 cells as well.

### Stabilization of p53 in response to ionizing radiation

Wild type p53 over-expression was assessed by RT-PCR and Western blotting. p53 levels significantly increased in H295R and SW-13 cell lines at 24 h after transfection with pBABE-neo-p53 vector and were maximum at 48 h. At 72 h from transfection, we observed a decrease in p53 levels, which seemed to be due to a reduction in *TP53* mRNA, probably because of the loss of transfected vectors (Figure S2).

However, in cells transfected with wild type p53 subjected to IR treatment, the effect on cell proliferation was strongest after 72 hours after transfection (48 h after irradiation). Therefore, expression levels of p53 were evaluated by RT-PCR and Western blotting in H295R, SW-13 and SK-OV-3 cells at the same time point.

**Figure 3:**
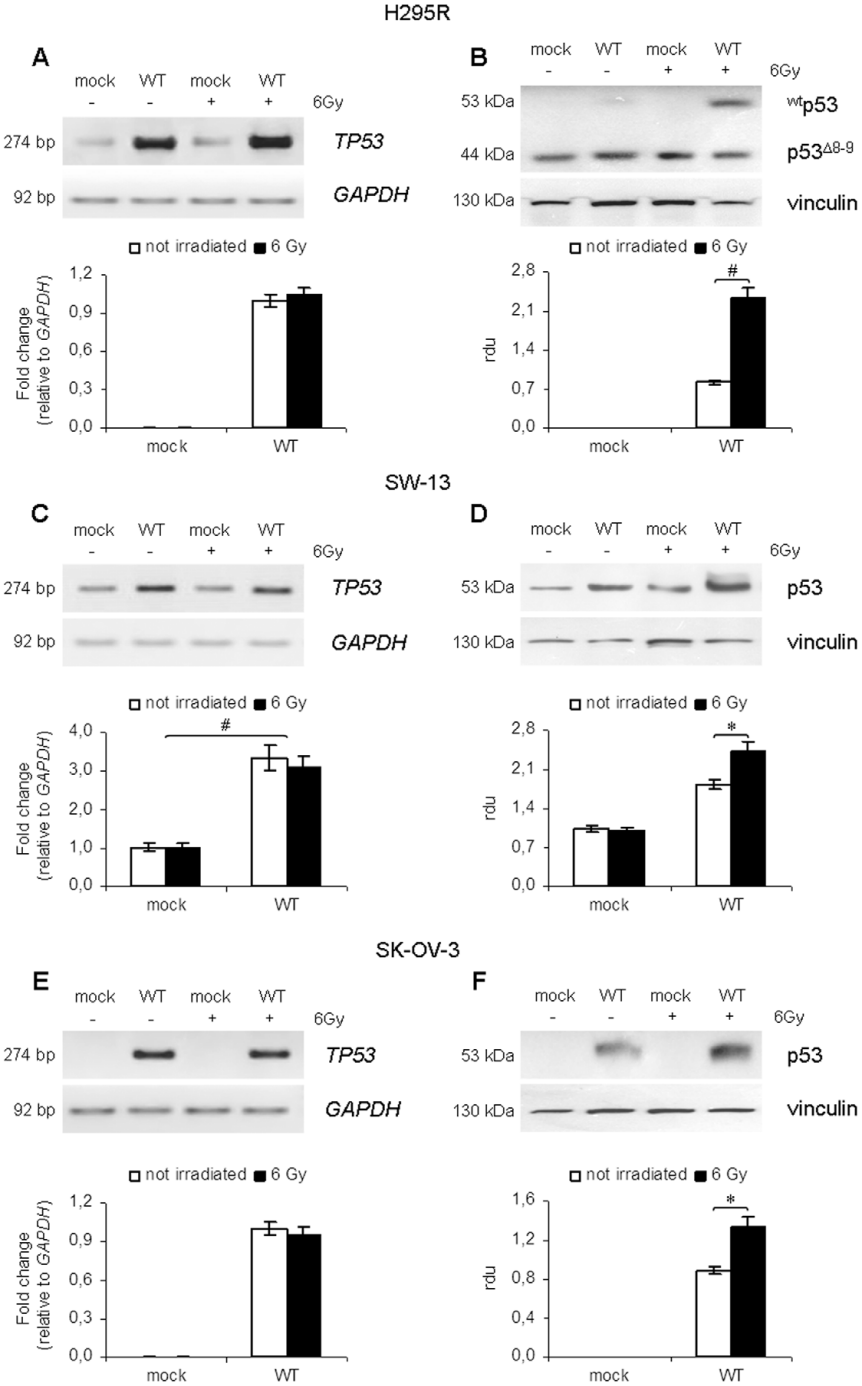
Effect of ionizing radiations on p53 stabilization. Expression levels of *TP53* and p53 protein evaluated by Real Time RT-PCR and Western blotting in H295R, SW-13 and SK-OV-3 cells 72 h after transfection with empty vector (mock) or p53-vector (WT). Samples were treated with IR at a dose of 6 Gy where indicated. (A) RT-PCR (top) and Real Time RT-PCR (bottom) analysis of *TP53* expression in H295R cells. Samples transfected with pBABE-neo-p53 vector show a 5-fold increase in *TP53* mRNA compared with mock. (B) Western blotting analysis of p53 in H295R cells (top) revealed a major band of ∼53 kDa (^wt^p53) and a minor band of ∼44 kDa, corresponding to the truncated form of p53 (p53^Δ8–9^). Densitometry shows that ^wt^p53 is stabilized by IR treatment (bottom). (C) RT-PCR analysis of *TP53* mRNA in SW-13 cells shows over-expression of the gene in samples transfected with pBABE-neo-p53 vector (top). Real Time RT-PCR analysis revealed a 3-fold increase in *TP53* transcript in samples transfected with p53 vector. The expression of *TP53* is not modulated by irradiation (bottom). (D) Levels of p53 protein in SW-13 cells significantly increase after IR treatment in cells transfected with pBABE-neo-p53 vector, while no change is detectable in samples transfected with empty vector. (E) In SK-OV-3 cells, no *TP53* transcript was detectable by RT-PCR in samples transfected with empty vector, while in cells transfected with pBABE-neo-p53 vector a strong signal, corresponding to *TP53* mRNA, is observable (top). Real Time RT-PCR analysis shows no modulation of *TP53* expression after irradiation (bottom). (F) Western blotting analysis of p53 in SK-OV-3 cells revealed a detectable band only in samples transfected with pBABE-neo-p53 vector (top). Densitometry shows that p53 levels increase after irradiation, indicating the stabilization of the protein (bottom). Results are representative of at least three independent experiments. *GAPDH* and vinculin were used for normalization. (*, p<0.05; #, p<0.01).

Real Time RT-PCR analysis confirmed that *TP53* gene is over-expressed in all samples transfected with pBABE-neo-p53 vector (Figure 3, panels A, C, E), either irradiated or not. Compared with mock, *TP53* mRNA was 5- and 3-fold more expressed in H295R and SW-13 cells, respectively. According to Yaginuma and Westphal [15], in SK-OV-3 cells, no *TP53* mRNA was detectable in mock samples, contrary to cells transfected with p53-vector.

Although *TP53* mRNA levels were higher in p53-transfected cells, they did not vary between WT irradiated and non-irradiated cells. On the contrary, p53 protein levels appeared to be significantly higher in p53-transfected cells subjected to IR treatment compared with the non-irradiated ones. In actual fact, in H295R cells the levels of wild type p53 increased 3 fold in irradiated WT cells compared with p53-transfected, non-irradiated cells (Figure 3, panel B). However, the endogenous form of p53 (p53^Δ8–9^) was not modulated by radiation treatment. SW-13 cells showed a 1,3-fold increase in p53 in irradiated WT samples compared to WT (Figure 3, panel D), whereas p53 was 1,5-fold higher in irradiated SK-OV-3 cells compared to untreated cells (Figure 3, panel F).

Treatment of H295R and SW-13 cells with different radiation doses (4, 6 and 8 Gy) confirmed that *TP53* mRNA expression levels were not influenced by IR (Figure S3, panels A, C). The administration of a radiation dose of 4 Gy did not induce a significant variation in p53 protein either. On the contrary, p53 was upregulated after the administration of radiation doses of 6 and 8 Gy; however, no significant changes were observed between these two doses (Figure S3, panels B, D), indicating that IR treatment at a dose of 6 Gy is sufficient to induce a stabilization of p53 in ACC cell lines.

The increase in p53 protein levels observed in irradiated samples is not due to an increase in *TP53* expression, since mRNA levels were not affected by radiation. This may be explained by an activation of p53 by IR, which translates in a stabilization of this protein only in cells subjected to this treatment. The stabilization of p53 may be considered predictive of its activation, thus explaining the effect on cell proliferation only observed in samples which received irradiation.

### TUNEL analysis of cell death

As p53 is a well-known inducer of the apoptotic process in response to genotoxic stresses [19], [20], we performed a TUNEL assay in order to analyse the presence of apoptosis in H295R, SW-13 and SK-OV-3 cell lines transfected with empty vector (mock) or p53-vector (WT), either irradiated or not.

**Figure 4:**
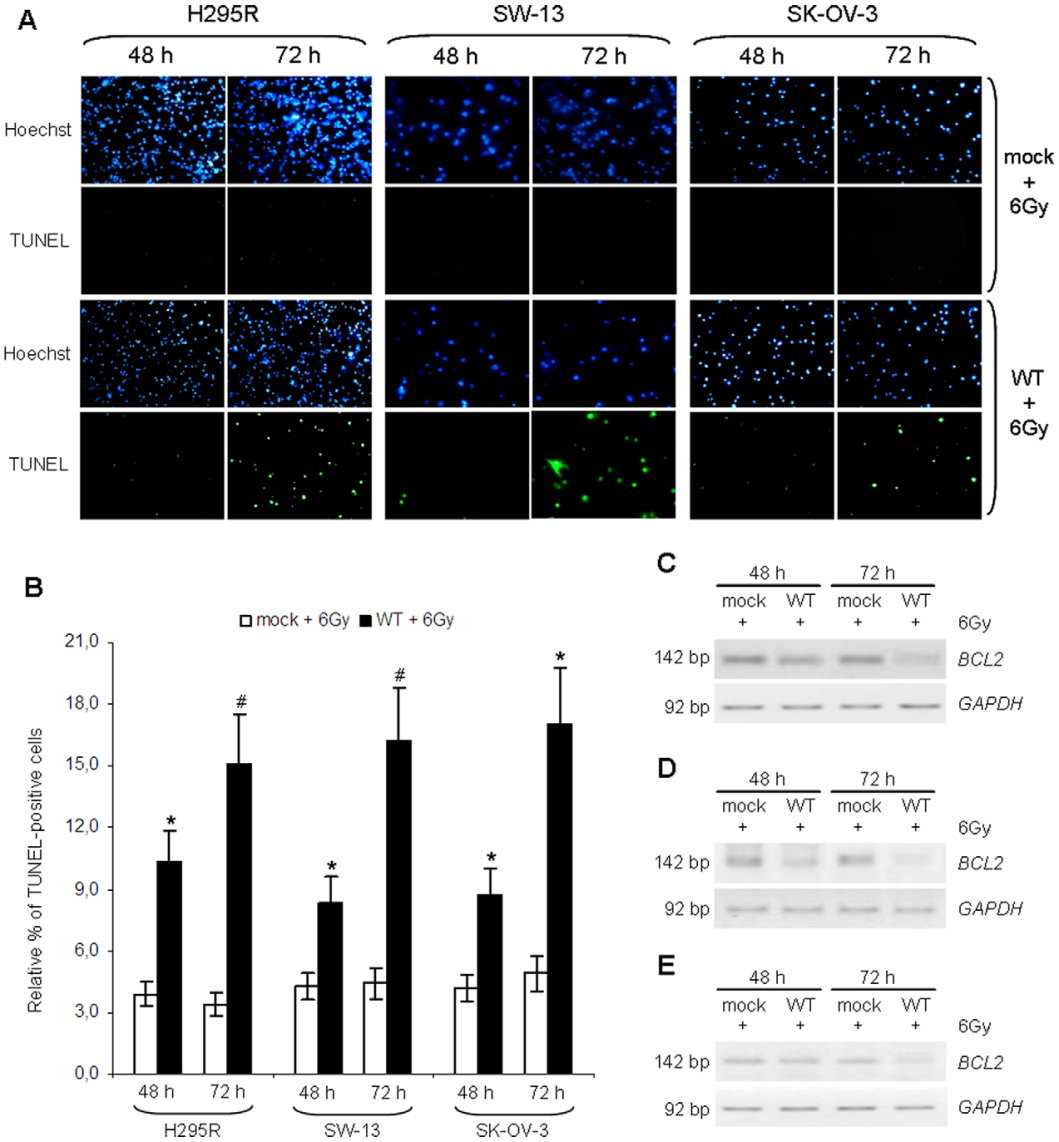
Induction of apoptosis by ionizing radiation following p53 stabilization. H295R, SW-13 and SK-OV-3 cell lines were transfected with empty vector (mock) or p53-vector (WT) and irradiated at a dose of 6 Gy. (A) TUNEL assay was performed at 48 and 72 after transfection. In all cell lines, TUNEL-positive cells are detectable only in irradiated samples expressing the wild type form of *TP53*. Cells were also stained with Hoechst 33342 and then visualized with a fluorescence microscope at a magnification of 40×. Figure shown is representative of three experiments performed in duplicate. (B) Percentage of apoptosis in H295R, SW-13 and SK-OV-3 was calculated by comparing TUNEL-positive cells with total cells, stained with Hoechst 33342. Apoptosis increases in cells transfected with p53 vector compared with mock-irradiated samples, starting from 48 h since transfection and the effect strengthens after 72 h. (*, p<0.05; #, p<0.01). (C) *BCL2* expression was evaluated in H295R cell line by RT-PCR. p53 restoration induces a significant reduction in *BCL2* expression at 48 and 72 h after irradiation. (D) Inverted images relative to semi-quantitative RT-PCR analysis of *BCL2* gene expression performed on SW-13 cells, showing a significant reduction in *BCL2* signal in cells transfected with p53-vector following ionizing radiations treatment. (E) The effect of p53 restoration on *BCL2* mRNA levels in response to irradiation was evaluated in SK-OV-3 cell lines. p53 inhibits *BCL2* expression and the effect is stronger at 72 h after transfection. Images are representative of at least three independent experiments. *GAPDH* was used for normalization.

As expected, no signs of apoptosis were detectable in samples not subjected to treatment with IR (data not shown). Apoptotic cell death was also absent in irradiated cells transfected with empty vector, whereas samples in which the wild type form of p53 was expressed appeared to be positive to TUNEL staining in all cell lines (Figure 4, panel A), indicating that the presence of a functional p53 is necessary for the induction of cell death in response to radiotherapy.

Apoptosis was detectable at 48 h after transfection of ^wt^p53 and increased at 72 h after transfection (48 h after irradiation) in all cell lines (Figure 4, panels A, B; Table S1). Apoptotic cell death increased by 2.6 fold at 48 h in irradiated H295R cells transfected with p53-vector compared with cells transfected with empty vector (10.3% *vs* 3.9%) (p<0.05) and by 4.4 fold at 72 h (15.1% *vs* 3.4%) (p<0.01) (Figure 4, panel B; Table S1). Less-differentiated SW-13 cells also showed a 2-fold increase in apoptosis at 48 h (8.4% *vs* 4.3%) (p<0.05) and a 3.7-fold increase at 72 h after transfection (16.3% *vs* 4.4%) (p<0.01) in irradiated WT sample compared with irradiated mock cells (Figure 4, panel B; Table S1). Similarly, transfection of empty vector did not greatly affect cell death in SK-OV-3 cell line either, while transfection of ^wt^p53 significantly increased the percentage of apoptotic cells in response to radiation treatment by 2 fold at 48 h (8.7% *vs* 4.2%) and by 3.5 fold at 72 h (17.1% *vs* 4.9%) (p<0.05) (Figure 4, panel B; Table S1).

Since many reports demonstrated that p53 is able to repress *BCL2* expression in response to an apoptotic stimulus [21]–[23], we analysed *BCL2* mRNA levels in cells treated with IR, either expressing the wild type form of *TP53* (WT) or not (mock), at 48 h and 72 h after transfection. RT-PCR analysis revealed that *BCL2* expression levels decreased in irradiated cells only when ^wt^p53 was present, whereas they held steady in samples transfected with empty vector. In accordance with TUNEL assay results, *BCL2* levels began to decrease starting from 48 h and minimum levels were observed at 72 h after transfection (Figure 4, panel C, D, E).

These data confirm that wild type p53 is required for the induction of cell death by apoptosis and down-regulation of *BCL2* in response to irradiation in H295R and SW-13 cells. Moreover, the absence of these effects in irradiated cells expressing only the endogenous mutant p53 seems to indicate that these mutants are non-functioning proteins are in no way influenced by IR treatment.

### Effect on *IGF2* expression

**Figure 5:**
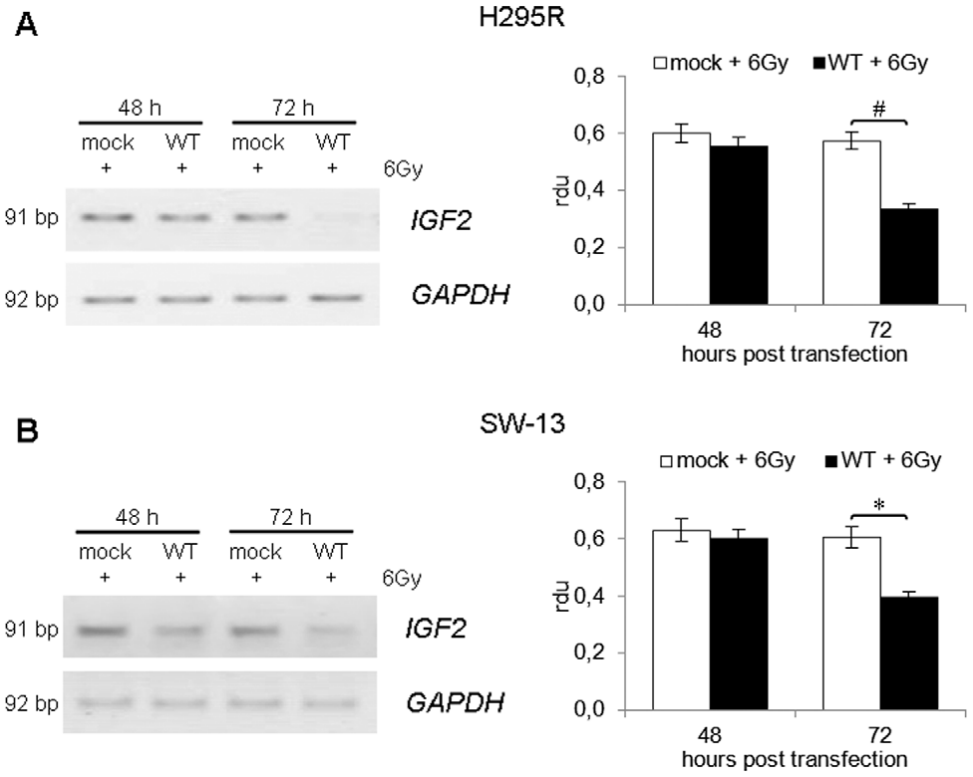
RT-PCR analysis of ^wt^p53 effect on *IGF2* expression. (A) Inverted images relative to semi-quantitative RT-PCR analysis of *IGF2* gene expression performed on H295R cells, showing a massive loss of *IGF2* signal in irradiated cells at 72 h after transfection of p53-vector (WT). (B) RT-PCR analysis of *IGF2* mRNA levels performed on SW-13 cells. A significant reduction in *IGF2* expression is detectable in cells transfected with p53-vector (WT) following ionizing radiations treatment. Images are representative of three independent experiments. *GAPDH* expression was used for normalization and bands' intensities were quantified using ImageJ. Band densitometry is shown in the right panels. (*, p<0.05; #, p<0.01).

IGF-II represents the main marker of ACC [24], [25] and its expression is tightly regulated by p53 [26], [27]. Since we demonstrated that ^wt^p53 is able to reduce cell proliferation and induce apoptosis in ACC cell lines in response to IR, we also investigated the effect of p53 activation on *IGF2* expression. RT-PCR analysis of *IGF2* mRNA expression in H295R cells (Figure 5, panel A) revealed that irradiation did not alter *IGF2* levels in samples transfected with empty vector. However, *IGF2* expression was reduced at 48 h after transfection (24 h after irradiation) and dramatically decreasing at 72 h after transfection (−42%; p<0.01) in cells expressing wild type p53.

Similar results were observed in the SW-13 cell line (Figure 5, panel B), although these cells express very low levels of *IGF2* mRNA. Even in this case, no effect on *IGF2* expression was observed after IR administration in cells expressing endogenous mutant p53, while a strong decrease in *IGF2* mRNA levels was observed in cells transfected with ^wt^p53 vector. This decrease was maximum at 72 h after transfection (−35%; p<0.05).

### Effect on Akt activation

Finally, we investigated the effect of IR on Akt activation in H295R and SW-13 cell lines either transfected with p53 vector (WT) or not (mock). This protein, in fact, is a main downstream effector of IGF-II signalling pathway and a key negative regulator of the apoptotic process.

**Figure 6:**
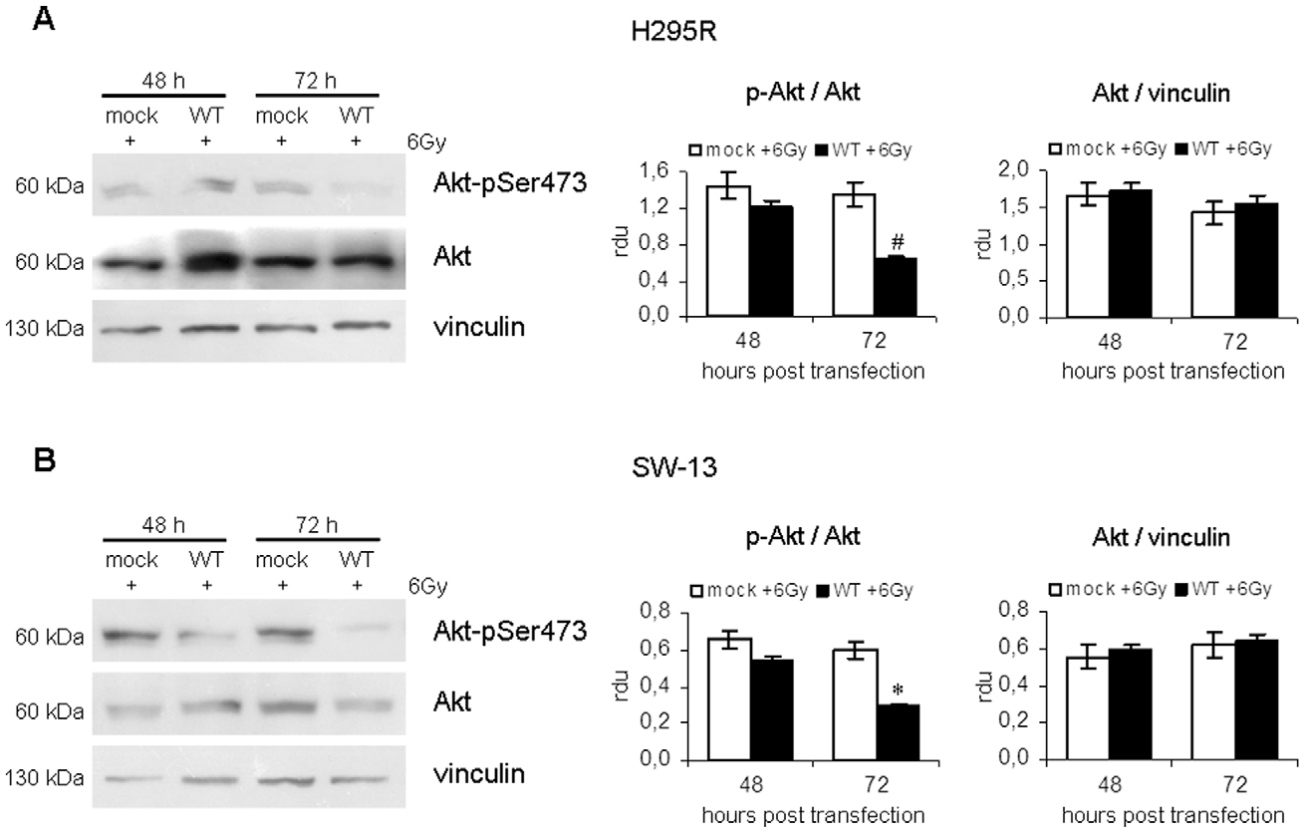
Effect of p53 stabilization on Akt activation after ionizing radiation treatment. (A) Western blotting analysis of total Akt and phosphorylated Akt levels, performed on irradiated H295R cells transfected with empty vector (mock) and p53-vector (WT) at 48 and 72 h after transfection. Densitometric analysis of Akt normalized to vinculin and p-Akt *versus* Akt shows that Akt expression is not influenced by p53 status, while the activated form of the protein is significantly reduced in ^wt^p53-expressing cells. (B) Total cell lysates obtained from SW-13 cell line were subjected to Western blotting analysis using antibodies targeting Akt and Akt-pSer473. Phosphorylation levels of Akt are significantly reduced by p53 stabilization in response to ionizing radiation treatment. Results shown are representative of at least three experiments. Bands' intensities were quantified using ImageJ and bands' densitometry is shown in the right panels. (*, p<0.05; #, p<0.01).

Western blotting analysis of total Akt and of its phosphorylated active form in H295R (Figure 6, panel A) and SW-13 (Figure 6, panel B) cells revealed that Akt was present in all samples and was not influenced by ^wt^p53 expression (data not shown). On the contrary, densitometric analysis of p-Akt normalized to total Akt showed that cells transfected with p53 vector displayed a marked decrease in Akt phosphorylation in response to IR compared with controls, meaning that p53 activation may be able to repress the activity of the anti-apoptotic protein Akt.

In accordance with our data concerning cell proliferation and apoptosis, the effect on Akt phosphorylation was evident at 48 h after transfection (−16% in H295R and −17% in SW-13 cells, respectively) and was maximum at 72 h after transfection (−53% in H295R and −51% in SW-13 cells; p<0.01).

## Discussion

ACC is an aggressive and heterogeneous malignancy and most of therapeutic options are often unsuccessful, thus indicating that alternative therapeutic strategies for ACC are needed [8], [9]. Reported toxicity of radiation therapy is mild to moderate, suggesting that radiotherapy may represent a valid choice. However, response rates to this treatment are highly variable. For a long time, ACC has been considered a radio-resistant tumour [2], [10], but over the last few years, many authors have demonstrated some effectiveness of radiotherapy [10], [11], [13], [28]. However, more studies are required to fully understand its real therapeutic potential. Recently, some indications about patient selection for radiotherapy of the tumour bed have been defined, based on resection status and histopathologic features of the neoplasm [11]. The importance of an individualized decision regarding radiotherapy has also been highlighted [10], [11].

Therefore, identification of other factors affecting radio-responsiveness may help the clinician in defining the best therapeutic approach. Extensive evidence over the last two decades reporting that p53 is required for efficacy of radiation therapy has generated considerable interest in developing strategies in tumours with defective p53 [17], [18], [29]. In fact, many reports have found a correlation between *TP53* mutations and radio-resistance in many cancer cell lines [30]–[33].

p53 function is frequently altered or lost in ACC. An association between *TP53* mutations and Li-Fraumeni syndrome has been found in more than 70% of families affected by this pathology, which means a higher risk of developing ACC, especially during childhood [4], [9]. Somatic mutations of *TP53* gene are often found in adult sporadic ACCs, with a frequency ranging from 20% to 70%, depending on the study. Moreover, LOH in 17p13 locus occurs in about 85% of cases of ACC, but not in benign adrenocortical adenomas [2], [9]. Although, these statistics strongly suggest an extensive role for *TP53* mutations in ACC onset, currently no data exist for the association between *TP53* status and radiotherapy efficacy in ACC. Therefore, we investigated the role of p53 protein in the response to this treatment in two cell lines derived from this tumour. The purpose was to evaluate the importance of p53 as a predictive factor for the outcome of radiation therapy, which might be useful for improving patients' prognosis and living conditions.

Our study was conducted on H295R and SW-13 cell models. H295R cells were obtained from a functional human ACC which have the ability to produce steroid hormones. In fact, they express steroidogenic enzymes CYP11A, HSD3B2, CYP11B1, CYP21, CYP17, CYP11B2, 3β-hydroxysteroid sulfotransferase, and aromatase (CYP19). They are also responsive to ACTH and Angiotensin-II stimulation [34]. On the contrary, the SW-13 cell line derives from a small cell carcinoma in the adrenal cortex. These cells are not able to produce steroids and their exact histopathologic origin has not been completely elucidated [34].

These cell lines, especially H295R, are typical of ACC and represent two useful models since they carry different mutations in the *TP53* gene. Deletion of exons 8 and 9 in H295R cells has previously been described by our group [13], while H193Y mutation in SW-13 cells was identified by Reincke *et al.* [14]. However, no data is available about the impact of these mutations on p53 function. A polymorphism in codon 72 (P72R) was also identified in the SW-13 cell line. The effect of this polymorphism on p53 activity has been widely debated [35]. A major susceptibility to cancer has been observed in homozygous carriers of the arginine allele and a reduction in disease-free and overall survival has been found in proline/arginine heterozygous patients with breast cancer [35], [36].

p53 mutant in H295R cells lacks part of the DNA binding domain and the entire C-terminal domain and SW-13 cells bare a homozygous point mutation in DBD, suggesting that p53 activity in these cell lines is completely lost.

We evaluated the effect of p53 restoration in ACC cell lines and in p53^−/−^ ovarian adenocarcinoma SK-OV-3 cells. It was previously demonstrated that lack of p53 is responsible for SK-OV-3 cells insensitivity to radiation treatment at low doses, while reintroduction of wild type p53 determines an effect on cell growth and viability in response to irradiation [32], [33]. Here, we demonstrate that transfection of SK-OV-3 cells with a construct harboring type *TP53* cDNA induces inhibition of cell proliferation and apoptosis in response to irradiation. Treatment of SK-OV-3 cells with a dose of 6 Gy induced a certain effect on cell proliferation even in samples transfected with empty vector. However, this effect was temporary since cells began to recover at 72 h after irradiation. Cell growth inhibition in irradiated cells was more relevant and long-lasting when ^wt^p53 was present. Moreover, cell death was only present in p53-expressing cells after irradiation, thus confirming that functional p53 is necessary for apoptotic response induced by irradiation.

We found that over-expression of wild type *TP53* is not able to induce a strong effect on cell proliferation or apoptosis in H295R and SW-13 cell lines. However, the presence of wild type p53 is required for inhibition of cell proliferation and induction of apoptotic cell death in response to IR treatment.

We noticed an increase in p53 levels after irradiation in our cell lines. This is due to the stabilization of the protein rather than to an enhanced transcription of the gene, suggesting its activation. In fact, p53, is maintained at low levels in resting cells and its stabilization in response to different stress stimuli represents a well-known mechanism which allows cells to preserve genome integrity after a DNA-damaging event. Inhibition of p53 function is mainly driven by Mdm2, which reduces the ability of p53 to activate gene expression and also participates in its degradation. Many post-translational modifications affecting both p53 and Mdm2 prevent p53 degradation, thus allowing this protein to promote cell cycle arrest, senescence and apoptosis [37], [38]. In our cell models, ^wt^p53 activity was also confirmed by the observation that only p53-transfected, irradiated samples were subjected to apoptosis, while non-irradiated and mock-transfected, irradiated cells were not. This indicates that the activation of wild type p53 is necessary for the induction of cell death following irradiation. Moreover, transcriptional activity of p53 was also enhanced, as was demonstrated by inhibition of the anti-apoptotic gene *BCL2*, whose expression is regulated by p53 [21]–[23]. *BCL2* levels were not influenced by irradiation in the absence of ^wt^p53, whereas restoration of p53 led to a strong decrease in its expression.

Hence, these findings confirm that stabilization of p53 following IR is necessary to induce apoptosis in ACC and SK-OV-3 cell lines. Moreover, our results shed new light on the functional characterization of H295R and SW-13 p53 mutants. In fact, when cells expressing the endogenous mutant forms of p53 were subjected to IR treatment, we observed no effect on apoptosis or *BCL2* expression, thus indicating that the mutations we identified cause a loss of the ability of p53 to induce apoptosis. Nevertheless, further studies would be required in order to fully characterize these mutants.

Considering these results, our findings also stress the importance of early ACC diagnosis, in order to undertake irradiation treatment during early stages of tumour development, virtually before the onset of mutations in the *TP53* gene. Moreover, new therapeutic approaches, based on mutant or inactive p53 re-activation by small molecules, like nutlins, PRIMA-1 and RITA [39], [40], associated with radiotherapy, may be considered. Furthermore, retrospective and prospective studies aiming at the evaluation of p53 status in patients who undergo radiotherapy may be very useful, in order to confirm the effective relationship between p53 activity and radio-responsiveness in ACC.

Finally, we investigated the ability of ^wt^p53 to repress *IGF2* expression. Insulin-like growth factor II (IGF-II) is over-expressed in more than 90% of ACCs [24], [41], [42] and represents the major marker that allows the distinction between benign adenomas and malignant tumours [24], [25]. Moreover, *IGF2* over-expression is considered a negative prognostic factor in ACC cases [2], [24]. Several studies demonstrated that p53 can inhibit *IGF2*, either directly, through regulation of *IGF2* P3 promoter activity, or indirectly, through the regulation of DNA methyltransferases (Dnmt) expression. This allows the maintenance of a correct methylation pattern of the imprinting control region (ICR) at the *IGF2*/*H19* loci [26], [27].

Here, we demonstrate that p53 activation in response to IR treatment is able to induce a strong effect on *IGF2* expression in ACC cell lines and especially in H295R cells, where we observed an almost complete suppression of *IGF2* levels. Moreover, we also evaluated the effect of IR administration on the anti-apoptotic protein Akt. It is one of the main downstream targets of IGF-II and was found to be hyper-activated in ACC [43], [44]. We observed a marked decrease in Akt activation levels in our cell lines, which is consistent with the induction of apoptosis seen in cells expressing ^wt^p53 subjected to irradiation. The activation of Akt through IGF-II/IGF-1R signaling is in fact involved in the regulation of many cell processes such as cell growth, division and survival. In particular, Akt mediates the inhibition of BAD (Bcl-2-associated agonist of cell death), thus stimulating Bcl-2 activity. Moreover, up-regulation of IGF-1R pathway is associated with increased radio-resistance through the down-regulation of pro-apoptotic factors Bax and Ku70/80, and the over-expression of anti-apoptotic proteins MVP (Major Vault Protein) and Bcl-2 [45], [46]. The ability of p53 to induce apoptosis and to inhibit Bcl-2, either directly or through repression of the IGF-II/Akt pathway, is therefore fundamental for cellular response to IR damage. The absence of these effects in irradiated H295R and SW-13 cells expressing only endogenous mutants of p53 clearly indicates that the wild type form of this protein is required to inhibit the aberrantly activated IGF-II/Akt signal transduction pathway in ACC.

Further studies are required to understand the exact mechanism leading to *IGF2* down-regulation by p53 after irradiation. However, the repression of IGF-II/Akt pathway activity represents an important therapeutic goal. As a matter of fact, several *in vitro* and *in vivo* studies demonstrated that over-expression of IGF-1R and aberrant activation of Akt are associated with increased radio-resistance and favor genetic instability after a radiation-induced DNA damage, by promoting non-homologous end joining (NHEJ) repair pathway over homologous recombination (HR). This happens through the repression of Ku70/80 protein and the over-expression of MVP [46]. The inhibitory effect of ^wt^p53 on IGF-II and Akt may thus promote tumour radio-responsiveness and prevent the onset of aberrant phenotypes due to non-specific recombination, which is one of the major limits to radiation-based therapies.

Furthermore, given the prognostic value of *IGF2* in ACC, inhibition of its expression by radiation-activated p53 may be helpful in reducing ACC aggressiveness, thus lessening the probability of disease recurrence. It may also increase disease-free and overall survival.

In conclusion, our study demonstrates that functional p53 is able to induce cell growth inhibition and apoptosis in response to radiotherapy in ACC cell lines, independently from their differentiation status and endocrine characteristics. This finding may be important for the integration of recommendations regarding radiotherapy practice suggested by Polat *et al.* [11], who believe that the decision on radiotherapy should be individualized in patients with localized tumours and R0 status. Therefore, we postulate that p53 status should be taken into consideration during therapeutic planning, since the presence of wild type p53 may represent a favorable predictive factor for radiotherapy in ACC cases.

## Materials and Methods

### Cell lines

Human adrenocortical carcinoma cell lines H295R and SW-13, ovarian adenocarcinoma cell line SK-OV-3 and prostate carcinoma-derived LNCaP cells were obtained from American Type Culture Collection (ATCC; LGC Standards Srl, Sesto San Giovanni, MI, Italy) and were cultured in the recommended media, according to standard mammalian tissue culture protocols and sterile technique.

H295R cells were grown in a culture medium consisting of a mixture of 1∶1 DMEM∶F12, SW-13 cells were cultured in Leibovitz's medium and SK-OV-3 and LNCaP cell lines were grown in RPMI medium (Lonza Group Ltd, Basel, Switzerland). All media were supplemented with 10% Australian-sourced foetal bovine serum, 2 mM L-Glutamine, 100 U/mL Penicillin and 100 µg/mL Streptomycin (Lonza). DMEM∶F12 medium was also enriched with ITS (Insulin-Transferrin-Selenium) (Sigma-Aldrich, St. Louis, MO, USA).

Cells were cultured in a 37°C incubator in an atmosphere of 5% CO_2_ in humidified air.

### *TP*53 sequencing

Total RNA of H295R and SW-13 cells was extracted using TRIzol Reagent (Invitrogen, Life Technologies Ltd, Paisley, UK), according to manufacturer's instructions and digested with DNAse I Amplification Grade (Invitrogen). Up to 200 ng of RNA were retro-transcribed using Omniscript RT Kit (Qiagen, Hilden, Germany) in a total volume of 20 µl, containing RT buffer 1×, 2 mM dNTPs, 0,5 µM oligo dT (Promega Corporation, Madison, WI, USA), 0,5 µM random hexamers (Promega), 10 U RNAse inhibitor (Promega) and 4 U Omniscript RT (Qiagen). 2 µl of the obtained cDNA were used for fragment amplification, using the following five primer pairs covering the whole coding sequence of *TP*53 gene:

Exons 2–4: *forward* 5′-GCCAGACTGCCTTCCGGGTC-3′, *reverse* 5′-GCCGGTGTAGGAGCTGCTGG-3′;

Exons 4–5: *forward* 5′-CCAGCAGCTCCTACACCGGC-3′, *reverse* 5′-GAGCAGCGCTCATGGTGGGG-3′;

Exons 5–7: *forward* 5′-CTGCTCAGATAGCATGGTCTG-3′, *reverse* 5′-TTGTAGTGGATGGTGGTACAGTCA-3′;

Exons 6–10: *forward* 5′-TGGTGCCCTATGAGCCGCCT-3′, *reverse* 5′-CGCTCACGCCCACGGATCTG-3′;

Exons 9–11: *forward* 5′-CCAGCTCCTCTCCCCAGCCA-3′, *reverse* 5′-GGGGGTGGGAGGCTGTCAGT-3′.

PCR products were purified using MICROCON Centrifugal Filter Devices (EMD Millipore, Billerica, MA, USA) and each fragment was sequenced using the Big Dye Terminator Cycle Sequence Ready Reaction kit on an ABI PRISM 310 Genetic Analyzer (Applied Biosystems, Life Technologies Corporation, Carlsbad, CA, USA), according to the manufacturer's protocol.

### Transfection and treatment

Cells were plated in 6-well plates, 80–90% confluent, cultured 24 h and then transiently transfected with empty vector (pBABE-neo) or p53 vector (pBABE-neo-p53), using FuGENE HD Transfection Reagent (Roche, Basel, Switzerland), according to manufacturer's instructions. pBABE vectors were kindly provided by Dr. Silvia Soddu and were chosen as they contain the Moloney murine leukemia virus (MMLV) Long Terminal Repeat, which has been proven to improve gene expression in a number of cell lines, compared with other internal promoters [16].

Cells were also co-transfected with a plasmid expressing Green Fluorescence Protein (pEGFP; Clontech, Saint-Germain-en-Laye, France). The ratio between pEGFP and pBABE vectors was 1∶9 and transfection efficiency was evaluated by direct visualization of GFP-expressing cells by fluorescence microscopy. An estimated transfection efficiency of ∼60% has been obtained.

24 h after transfection, cells were irradiated by a Varian Clinac 600 c/d 6MV photon beam with a single radiation dose of 6 Gy. In order to test the dose of choice, lower doses (4 Gy) and higher doses (8 Gy) were administered. Further analysis was performed until 72 h post transfection (48 h after irradiation).

Cell proliferation was evaluated by cell count using a haemocytometer (Sigma-Aldrich). The presence of necrosis following radiation treatment was assessed by trypan blue staining. After trypsinization, cells were suspended in DPBS 1× and mixed with equal volume of trypan blue 0,4% (Lonza), and the percentage of stained cells was then determined.

### TUNEL assay

After transfection and irradiation, total cells were harvested and washed in DPBS 1×. Nearly 100.000 cells *per* sample were centrifuged on poly-L-lysine coated coverslips and then fixed with 4% formaldehyde for 20 minutes at room temperature. Apoptosis was assessed using *In Situ* Cell Death Detection Kit, POD (Roche), according to manufacturer's instructions. Positive controls were incubated with 300 U DNAse for 10 minutes at room temperature. Samples were also stained with Hoechst 33342 (Sigma-Aldrich), diluted 50 ng/mL in methanol, by incubation at 37°C for 10 minutes. Fluorescence of cells was detected using an epifluorescence microscopy (Leica Microsystems Srl, Milan, Italy), using channels able to detect green fluorescence, which is typical of TUNEL-positive cells (540 nm) and blue fluorescence, emitted by Hoechst 33342 (455 nm). At least ten randomly chosen fields *per* sample were photographed and subsequently analysed using ImageJ software (Image Processing and Analysis in Java, version 1.39 u). Percentage of apoptosis was determined by comparing TUNEL-positive cells with total, Hoechst-stained cells. Significant differences between samples were evaluated using ANOVA and Student's *t* tests.

### RT-PCR and Real Time RT-PCR analysis

RNA of H295R, SW-13 and SK-OV-3 cells was prepared as described in the *TP53 sequencing* paragraph. RT-PCR analysis of *TP53*, *BCL2* and *IGF2* expression were carried out in a 50 µl reaction volume containing 2 µl of cDNA, using the following primers:

*TP53 ex 2–4*: *forward*: 5′-GCCAGACTGCCTTCCGGGTC-3′; *reverse*: 5′-GCCGGTGTAGGAGCTGCTGG-3′;

*TP53 ex 9*: *forward*: 5′-CCAGCTCCTCTCCCCAGCCA-3′; *reverse*: 5′-CGCTCACGCCCACGGATCTG-3′;

*BCL2*: *forward*: 5′-ATGACTGAGTACCTGAACCG-3′; *reverse*: 5′-CCAAACTGAGCAGAGTCTTC-3′;

*IGF2*: *forward*: 5′-GTTTGCGACACGCAGCA-3′; *reverse*: 5′-AAGCACCAGCATCGACTT-3′;

*GAPDH*: *forward*: 5′-TCTTTTGCGTCGCCAGCCGA-3′; *reverse*: 5′-ACCAGGCGCCCAATACGACC-3′.

Real Time RT-PCR analysis was performed on a iCycler iQ instrument (Bio-Rad Laboratories, Hercules, CA, USA). *TP53* mRNA expression levels were analysed using 25 µl of iQ SYBR Green Supermix (Bio-Rad Laboratories) and 2 µl of cDNA in a final volume of 50 µl. Samples were analysed in triplicate and relative quantification was performed by analysing the expression levels of the endogenous control *GAPDH*. The relative changes in *TP53* gene expression were calculated with the 2^−ΔΔCt^ method [47].

### Western blotting analysis

After medium removal, cells were washed and scraped into PBS. Total protein extracts were obtained by suspension of cellular pellets with 70 µl of extraction buffer, sonication on ice and centrifugation at 20000×g. Protein concentrations were determined by Bradford colorimetric assay (Bio-Rad Laboratories). SDS-PAGE on 10% polyacrylamide gel was performed using 50 µg of protein extracts and proteins were then transferred on a nitrocellulose membrane. Membrane was blocked for 1 hour with 5% non-fat dry milk in DPBS 1× containing 0,1% Tween 20 (Bio-Rad Laboratories) at room temperature and then incubated with specific antibodies overnight at +4°C. The antibodies used were p53(DO-1) 1∶500 (Santa Cruz Biotechnology, CA, USA), cyclin E 1∶250 (BD Biosciences, Franklin Lakes, NJ USA), cyclin D1 1∶250 (BD Biosciences), Akt-pSer473 1∶400 (Santa Cruz Biotechnology), Akt 1,2,3 1∶100 (Santa Cruz Biotechnology), vinculin 1∶1000 (Sigma-Aldrich). The visualization of the antigens was done with enhanced chemiluminescence (ECL) detection reagents (Thermo Fisher Scientific Inc., Rockford, IL, USA) after incubation with a goat anti-mouse or goat anti-rabbit secondary antibody (Sigma-Aldrich) for 1 hour at room temperature. Quantitative analysis was performed using ImageJ software.

### Statistical analysis

All values obtained are means of at least three independent experiments performed in duplicate. Results are presented as mean value ± S.D. (standard deviation). Control and treated groups were compared using the analysis of variance (ANOVA) test; in addition, a comparison of individual treatments was also performed, using Student's *t* test. In all analyses, a p-value of <0.05 was considered statistically significant. Data were processed using Assistat (version 7.6b) and Microsoft Excel software.

## Supporting Information

Figure S1 **Expression levels of p53 in SW-13 and LNCaP cell lines.** Western blotting analysis of p53 levels in SW-13 adrenocortical carcinoma cell line (mutated p53) and LNCaP prostate carcinoma cells (wild type p53), showing a 4-fold higher expression of p53 in SW-13 cells. Bands' intensities were quantified using ImageJ, and vinculin was used for normalization (#, p<0.01).
(TIF)

Figure S2 **Over-expression of p53 in SW-13 and H295R ACC cell lines after transfection with pBABE-neo-p53 vector.** Expression levels of p53 were evaluated by RT-PCR and Western blotting in H295R and SW-13 cells at 24, 48 and 72 h after transfection with empty vector (mock) or p53-vector (WT). (A) RT-PCR analysis of *TP53* expression in H295R cell line, showing a significant increase in mRNA levels after transfection with pBABE-neo-p53 vector starting from 24 h and decreasing at 72 h. (B) Western blotting analysis of p53 expression shows expression of wild type p53 after transfection with pBABE-neo-p53 vector (WT). Protein levels are detectable at 24 h after transfection and increase at 48 h, while a significant decrease is observable at 72 h. (C) Inverted images relative to semi-quantitative RT-PCR analysis of *TP53* gene expression in SW-13 cell line. mRNA levels increase at 24 h after transfection with p53-vector (WT) and are maximum at 48 h. (D) Western blotting analysis performed on SW-13 total lysates revealed over-expression of p53 protein in samples transfected with pBABE-neo-p53 vector (WT) compared with those transfected with empty vector. p53 levels increase until 48 h, then lower at 72 h. Results are representative of at least three independent experiments. Bands' intensities were quantified with ImageJ software using *GAPDH* and vinculin for normalization. (*, p<0.05; #, p<0.01).
(TIF)

Figure S3 **Dose-dependent effect of ionizing radiations on p53 stabilization.** H295R and SW-13 cells were transfected with empty vector (mock) or p53-vector (WT) and then untreated (−IR) or treated with different doses of ionizing radiations (4, 6 and 8 Gy). Expression levels of p53 were evaluated by RT-PCR and Western blotting at 72 h after transfection (48 h after irradiation). (A) Inverted images relative to semi-quantitative RT-PCR analysis of *TP53* gene expression in H295R cell line. A significant increase in mRNA levels is observable in all samples transfected with pBABE-neo-p53 vector (WT), while irradiation at different doses did not influence *TP53* levels. (B) Western blotting analysis of p53 in H295R cells shows not significant variation in ^wt^p53 expression after irradiation at a dose of 4 Gy, while ionizing radiation (IR) treatment with higher doses significantly induces p53 accumulation, indicating protein stabilization. (C) RT-PCR analysis of *TP53* expression in SW-13 cell line shows mRNA over-expression in samples transfected with p53-vector (WT) compared with those transfected with empty vector (mock). No significant change in *TP53* levels is observable after IR treatment. (D) The effect of IR on p53 protein levels was evaluated by Western blotting in SW-13 cell line. Low doses (4 Gy) did not induce significant changes in p53 expression compared with non-irradiated control (−IR). At higher dose (6 and 8 Gy), no effect was observed in samples transfected with empty vector (mock), while there is a significant increase in p53 levels in cells transfected with pBABE-neo-p53 (WT), consisting with the stabilization of wild type p53. Results are representative of at least three independent experiments. Bands' intensities were quantified with ImageJ software using *GAPDH* and vinculin for normalization. (*, p<0.05; #, p<0.01).
(TIF)

Table S1 **TUNEL analysis after wt p53 over-expression and irradiation.** H295R, SW-13 and SK-OV-3 cell lines were transfected with empty vector (mock) or p53- vector (WT) and subjected to ionizing radiation treatment at a dose of 6 Gy. Percentage of apoptosis was evaluated at 48 and 72 h after transfection (24 and 48 h from irradiation), by comparing TUNEL positive cells with Hoechst-positive, total, cells using ImageJ software.
(DOC)
