# Supplementary figures and images for: p53 Stabilization Induces Cell Growth Inhibition and Affects IGF2 Pathway in Response to Radiotherapy in Adrenocortical Cancer Cells
Source: PLoS One. 2012 Sep 19;7(9):e45129. doi: 10.1371/journal.pone.0045129 (PMC3446967; doi:10.1371/journal.pone.0045129)

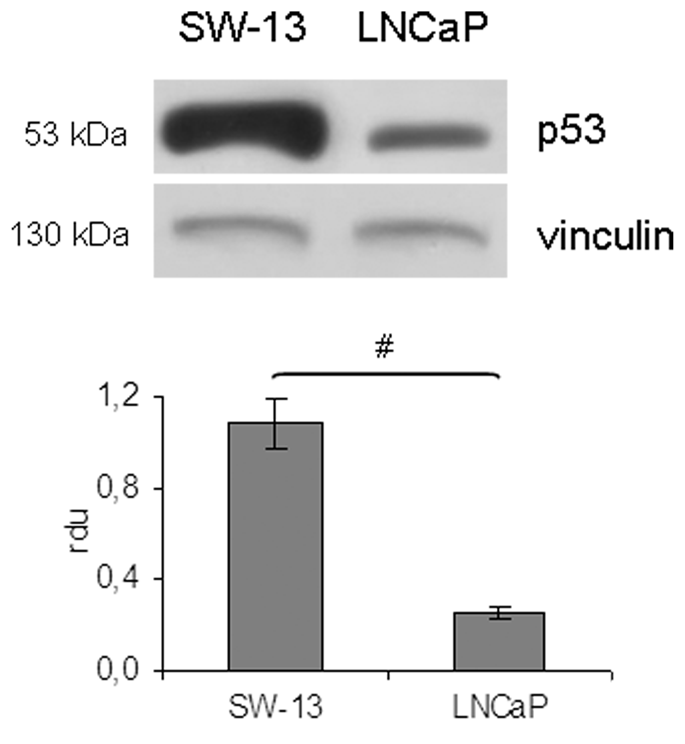

Supplement: Figure S1 — Expression levels of p53 in SW-13 and LNCaP cell lines. Western blotting analysis of p53 levels in SW-13 adrenocortical carcinoma cell line (mutated p53) and LNCaP prostate carcinoma cells (wild type p53), showing a 4-fold higher expression of p53 in SW-13 cells. Bands' intensities were quantified using ImageJ, and vinculin was used for normalization (#, p<0.01). (TIF) [file pone.0045129.s001.tif]

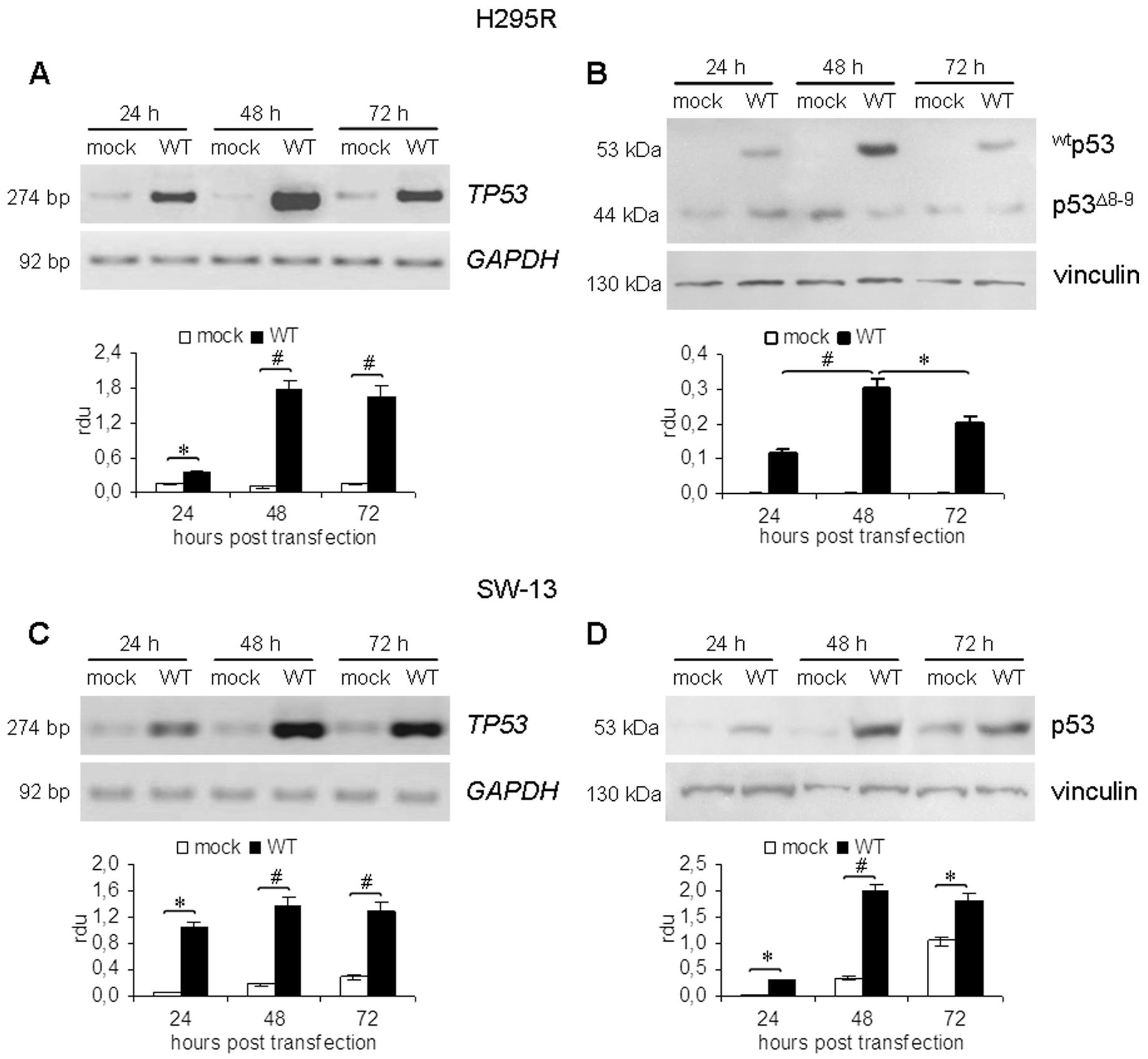

Supplement: Figure S2 — Over-expression of p53 in SW-13 and H295R ACC cell lines after transfection with pBABE-neo-p53 vector. Expression levels of p53 were evaluated by RT-PCR and Western blotting in H295R and SW-13 cells at 24, 48 and 72 h after transfection with empty vector (mock) or p53-vector (WT). (A) RT-PCR analysis of TP53 expression in H295R cell line, showing a significant increase in mRNA levels after transfection with pBABE-neo-p53 vector starting from 24 h and decreasing at 72 h. (B) Western blotting analysis of p53 expression shows expression of wild type p53 after transfection with pBABE-neo-p53 vector (WT). Protein levels are detectable at 24 h after transfection and increase at 48 h, while a significant decrease is observable at 72 h. (C) Inverted images relative to semi-quantitative RT-PCR analysis of TP53 gene expression in SW-13 cell line. mRNA levels increase at 24 h after transfection with p53-vector (WT) and are maximum at 48 h. (D) Western blotting analysis performed on SW-13 total lysates revealed over-expression of p53 protein in samples transfected with pBABE-neo-p53 vector (WT) compared with those transfected with empty vector. p53 levels increase until 48 h, then lower at 72 h. Results are representative of at least three independent experiments. Bands' intensities were quantified with ImageJ software using GAPDH and vinculin for normalization. (*, p<0.05; #, p<0.01). (TIF) [file pone.0045129.s002.tif]

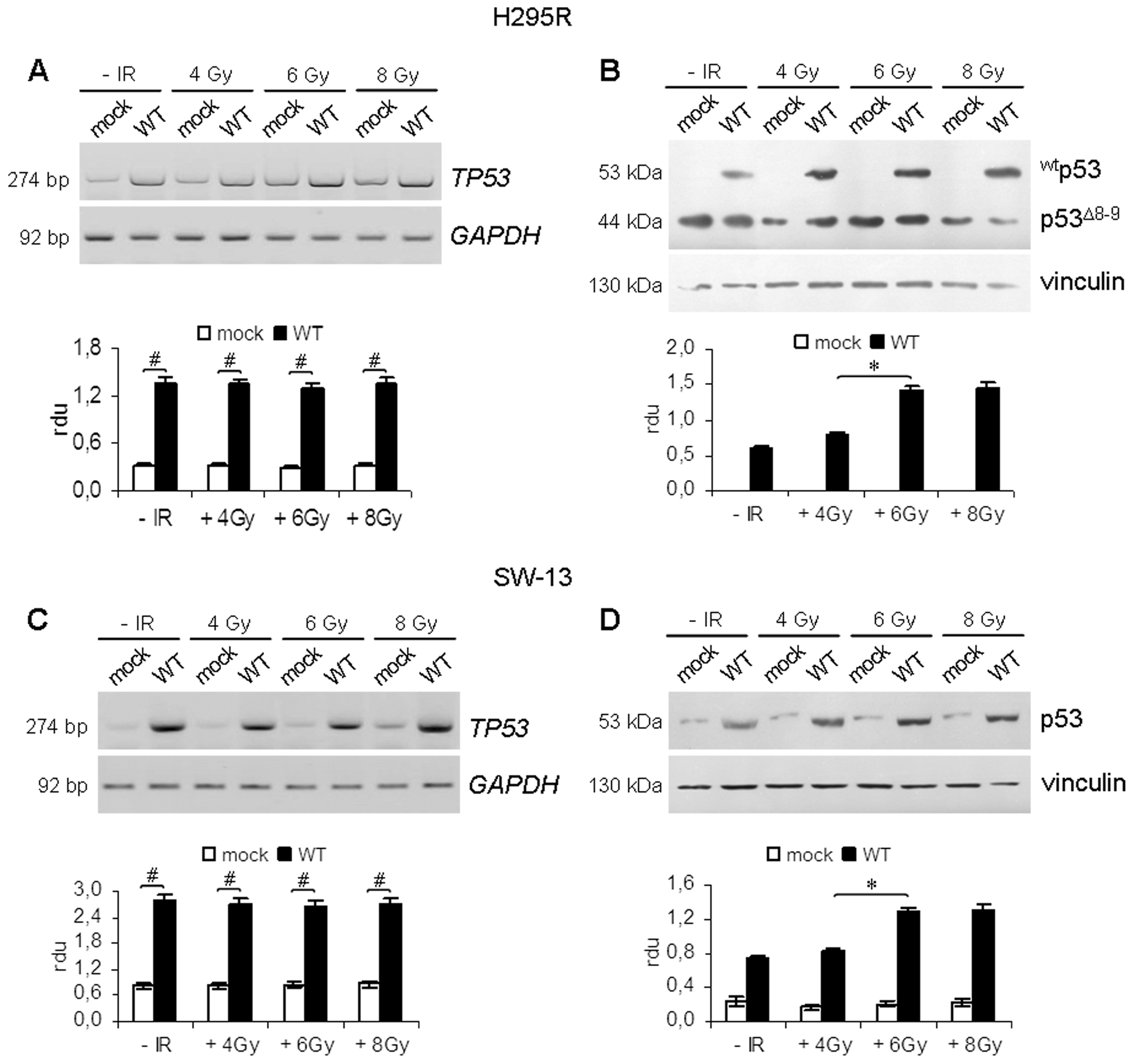

Supplement: Figure S3 — Dose-dependent effect of ionizing radiations on p53 stabilization. H295R and SW-13 cells were transfected with empty vector (mock) or p53-vector (WT) and then untreated (−IR) or treated with different doses of ionizing radiations (4, 6 and 8 Gy). Expression levels of p53 were evaluated by RT-PCR and Western blotting at 72 h after transfection (48 h after irradiation). (A) Inverted images relative to semi-quantitative RT-PCR analysis of TP53 gene expression in H295R cell line. A significant increase in mRNA levels is observable in all samples transfected with pBABE-neo-p53 vector (WT), while irradiation at different doses did not influence TP53 levels. (B) Western blotting analysis of p53 in H295R cells shows not significant variation in wtp53 expression after irradiation at a dose of 4 Gy, while ionizing radiation (IR) treatment with higher doses significantly induces p53 accumulation, indicating protein stabilization. (C) RT-PCR analysis of TP53 expression in SW-13 cell line shows mRNA over-expression in samples transfected with p53-vector (WT) compared with those transfected with empty vector (mock). No significant change in TP53 levels is observable after IR treatment. (D) The effect of IR on p53 protein levels was evaluated by Western blotting in SW-13 cell line. Low doses (4 Gy) did not induce significant changes in p53 expression compared with non-irradiated control (−IR). At higher dose (6 and 8 Gy), no effect was observed in samples transfected with empty vector (mock), while there is a significant increase in p53 levels in cells transfected with pBABE-neo-p53 (WT), consisting with the stabilization of wild type p53. Results are representative of at least three independent experiments. Bands' intensities were quantified with ImageJ software using GAPDH and vinculin for normalization. (*, p<0.05; #, p<0.01). (TIF) [file pone.0045129.s003.tif]
