# Supplementary material for: p53 Stabilization Induces Cell Growth Inhibition and Affects IGF2 Pathway in Response to Radiotherapy in Adrenocortical Cancer Cells
Source: PLoS One. 2012 Sep 19;7(9):e45129. doi: 10.1371/journal.pone.0045129 (PMC3446967; doi:10.1371/journal.pone.0045129)
Supplement: Table S1 — TUNEL analysis after wt p53 over-expression and irradiation. H295R, SW-13 and SK-OV-3 cell lines were transfected with empty vector (mock) or p53- vector (WT) and subjected to ionizing radiation treatment at a dose of 6 Gy. Percentage of apoptosis was evaluated at 48 and 72 h after transfection (24 and 48 h from irradiation), by comparing TUNEL positive cells with Hoechst-positive, total, cells using ImageJ software. (DOC) [file pone.0045129.s004.doc]

**Table S1**

| **sample** | **n° of Hoechst-positive cells** | **n° of TUNEL-positive cells** | **% of apoptosis** |
| --- | --- | --- | --- |
| *H295R* | | | |
| mock 48 h | 4017 | 88 | 2,19 |
| WT 48 h | 3078 | 105 | 3,40 |
| mock + 6Gy 48 h | 3087 | 120 | 3,89 |
| WT + 6Gy 48 h | 2132 | 219 | 10,27 |
| mock 72 h | 3969 | 128 | 3,22 |
| WT 72 h | 4083 | 132 | 3,23 |
| mock + 6Gy 72 h | 3364 | 114 | 3,39 |
| WT + 6Gy 72 h | 3465 | 524 | 15,12 |
| *SW-13* | | | |
| mock 48 h | 2349 | 88 | 3,75 |
| WT 48 h | 2280 | 95 | 4,17 |
| mock + 6Gy 48 h | 2241 | 96 | 4,28 |
| WT + 6Gy 48 h | 2238 | 187 | 8,36 |
| mock 72 h | 3870 | 158 | 4,08 |
| WT 72 h | 3851 | 179 | 4,65 |
| mock + 6Gy 72 h | 3820 | 169 | 4,42 |
| WT + 6Gy 72 h | 3778 | 614 | 16,25 |
| *SK-OV-3* | | | |
| mock 48 h | 1529 | 51 | 3,34 |
| WT 48 h | 1490 | 61 | 4,09 |
| mock + 6Gy 48 h | 1036 | 43 | 4,15 |
| WT + 6Gy 48 h | 1010 | 88 | 8,71 |
| mock 72 h | 2184 | 77 | 3,53 |
| WT 72 h | 2188 | 94 | 4,30 |
| mock + 6Gy 72 h | 2198 | 108 | 4,91 |
| WT + 6Gy 72 h | 2014 | 344 | 17,08 |

**Table S1. TUNEL analysis after wtp53 over-expression and irradiation.**

H295R, SW-13 and SK-OV-3 cell lines were transfected with empty vector (mock) or p53-vector

(WT) and subjected to ionizing radiation treatment at a dose of 6 Gy. Percentage of apoptosis was

evaluated at 48 and 72 h after transfection (24 and 48 h from irradiation), by comparing TUNEL positive cells with Hoechst-positive, total, cells using ImageJ software.
